# Transcriptomic profiles of aging in naïve and memory CD4^+^ cells from mice

**DOI:** 10.1186/s12979-017-0092-5

**Published:** 2017-06-20

**Authors:** Jackson Taylor, Lindsay Reynolds, Li Hou, Kurt Lohman, Wei Cui, Stephen Kritchevsky, Charles McCall, Yongmei Liu

**Affiliations:** 1 Department of Epidemiology & Prevention, Public Health Sciences, Wake Forest School of Medicine, Winston-Salem, NC 27157 USA; 2 Department of Internal Medicine, Wake Forest School of Medicine, Winston-Salem, NC 27157 USA; 3 Present Address: Department of Molecular Biology, Cell Biology, Biochemistry at Brown University, Providence, RI 02912 USA

**Keywords:** Aging, T cells, CD4+, Transcriptomic, NFKB, Enhancer, Inflammation

## Abstract

**Background:**

CD4+ T cells can be broadly divided into naïve and memory subsets, each of which are differentially impaired by the aging process. It is unclear if and how these differences are reflected at the transcriptomic level. We performed microarray profiling on RNA derived from naïve (CD44^low^) and memory (CD44^high^) CD4+ T cells derived from young (2–3 month) and old (28 month) mice, in order to better understand the mechanisms of age-related functional alterations in both subsets. We also performed follow-up bioinformatic analyses in order to determine the functional consequences of gene expression changes in both of these subsets, and identify regulatory factors potentially responsible for these changes.

**Results:**

We found 185 and 328 genes differentially expressed (FDR ≤ 0.05) in young vs. old naïve and memory cells, respectively, with 50 genes differentially expressed in both subsets. Functional annotation analyses highlighted an increase in genes involved in apoptosis specific to aged naïve cells. Both subsets shared age-related increases in inflammatory signaling genes, along with a decrease in oxidative phosphorylation genes. Cis-regulatory analyses revealed enrichment of multiple transcription factor binding sites near genes with age-associated expression, in particular NF-κB and several forkhead box transcription factors. Enhancer associated histone modifications were enriched near genes down-regulated in naïve cells. Comparison of our results with previous mouse and human datasets indicates few overlapping genes overall, but suggest consistent up-regulation of *Casp1* and *Il1r2*, and down-regulation of *Foxp1* in both mouse and human CD4+ T cells.

**Conclusions:**

The transcriptomes of naïve and memory CD4+ T cells are distinctly affected by the aging process. However, both subsets exhibit a common increase inflammatory genes and decrease in oxidative phosphorylation genes. NF-κB, forkhead box, and Myc transcription factors are implicated as upstream regulators of these gene expression changes in both subsets, with enhancer histone modifications potentially driving unique changes unique to naïve cells. Finally we conclude that there is little overlap in age-related gene expression changes between humans and mice; however, age-related alterations in a small subset of genes may be conserved.

**Electronic supplementary material:**

The online version of this article (doi:10.1186/s12979-017-0092-5) contains supplementary material, which is available to authorized users.

## Background

T cells are critical mediators of the body’s immune response to infection and tumor formation. Advanced age is linked to a number of functional impairments in human T cells [1], many of which are also observed in laboratory animals [2]. The primary consequence of age-related decline in T cell function is an increased risk of mortality from infection in elderly individuals, which stems from both an impaired adaptive immune response [1, 2] and a decreased effectiveness of vaccination [3]. Impaired function of aged T cells may also contribute to increased incidence of cancer in the elderly [1]. Further, T cells are thought to exhibit increased autoimmune activity with age [4], which contributes to chronic inflammatory disorders such as rheumatoid arthritis. The ability to prevent or reverse age-related changes in T cells is therefore of great importance for the treatment of human disease. However the molecular underpinnings of age-related functional impairment in T cells are not fully understood.

T cells exist in a variety of subsets, which are classified according to function and surface protein expression. The two major classes are CD4^+^ (helper) and CD8^+^ (cytotoxic) T cells-both of which are further divided into naïve (never exposed to cognate antigen) and memory (previously exposed to cognate antigen) subsets. A number of well-characterized aging phenotypes have been observed in general T cell populations including: decreased proportion of naïve T cells [2], decreased proliferation in response to antigen stimulation [5], altered apoptotic signaling [6, 7], decreased T cell receptor (TCR) diversity [8], and altered cytokine production [9]. Naïve and memory subsets are also differentially affected by the aging process. For example, memory CD4^+^ T cells do not exhibit age-related impairment in cytokine-mediated proliferation, while naïve CD4^+^ T cells do [10]. In addition, memory cells generated from young naïve cells function well even into old age, while memory cells generated from aged naïve cells function poorly [9]. The turnover rate and replicative capacity of both subsets is also different. Naïve T cells have a 10-fold lower turnover rate than memory [10] and also possess longer telomeres [11] - which allows naïve T cell to divide a far greater number of times than memory cells before entering replicative senescence. In addition, the lifespan of naïve CD4^+^ cells increases with age in mice and this enhanced longevity has been proposed to cause functional deficits during the aging process [7].

What underlies these general and subset-specific aging phenotypes in CD4+ T cells? A probable driving force is changes in gene expression. A number of individual genes have been demonstrated to change expression levels between young and aged T cells. Perhaps the most consistent finding in T cells (both CD4+ and CD8+) is age-related loss of the co-stimulatory surface protein CD28, which is attributed to diminished transcription of the CD28 gene [12], leading to reduced TCR diversity and antigen-induced proliferation. Additionally, transcript expression of the tumor suppressor *p16* ^*INK4a*^ show a positive correlation with donor age in human CD4+ T cells [13], which is associated with increased IL-6 expression. The functional consequence of increased *p16* ^*INK4a*^ expression with age is unclear but it appears to be a useful predictor of chronological age and may be connected to clinical markers of frailty and cellular senescence. Decline in expression of the microRNA miR-181a in human CD4+ T cells leads to increased expression of DUSP6, which impairs ERK signaling and subsequently impairs T cell activation, proliferation, and differentiation [14].

Whole-transcriptome profiling with microarray and RNA-seq technologies has allowed a more in depth look at the molecular basis of T cell aging. Widespread alteration of mRNA expression levels is a hallmark of T cell aging in mice and humans [15], with changes in specific genes providing a logical source for some of the observed age-related phenotypes. An initial microarray study of age-related changes in mouse CD4^+^ T cells found that aging was associated with increased expression of multiple chemokine receptor gene transcripts [16]-a finding that was confirmed in a later study [17]. An age-related decrease in expression of several cell cycle genes with pro-proliferative function has also been reported from microarray analysis of young and aged T cells from mice [17, 18]. Further, increased mRNA expression of both pro- and anti-apoptotic genes has also been reported [17], which may underlie the complex changes in apoptotic signaling observed in aged T cells [6, 7, 19]. In humans, a previous transcriptomic profiling of young and old CD4^+^ T cells revealed an enrichment of genes induced by NF-κB that were up-regulated in aged individuals [20]. Our group recently performed global gene expression profiling on purified CD4^+^ T cells and CD14^+^ monocytes from a large human cohort, aged 55–91 [21]. In CD4^+^ T cells, we found suggestive evidence for enrichment for immune function amongst gene transcripts up-regulated with age and enrichment for ribonucleoprotein complex involvement in genes down-regulated with age.

Although our results and those from others offer a molecular basis for some of the more general phenotypes observed during aging in CD4+ T cells, they did not compare individual subsets and are unable to offer insight into gene expression changes which may underlie subset-specific age-related phenotypes. We sought to determine to what degree age-related transcriptomic changes in CD4+ T cells were unique to naïve and memory subsets, respectively, and whether these changes could be linked to their respective phenotypes. To this end, we utilized whole-genome microarray analyses to identify transcriptomic changes that occur during aging in naïve and memory CD4^+^ populations. Using these data, we also performed comprehensive bioinformatic analyses in order to elucidate biological consequences of altered gene expression and identify up-stream cis-regulators of age-affected genes. Finally, we compared our results in mouse with previous published mouse and human data sets to identify key genes which show conserved and reproducible alterations during aging. Our results identify molecular targets which may drive age-related functional decline in naïve and memory CD4+ cells and suggest some of these targets are conserved in humans.

## Results

**Fig. 1:**
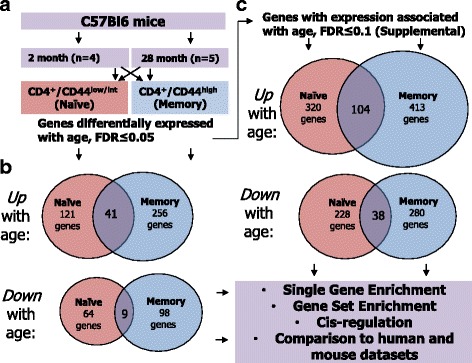
Overview of experimental design and results. **a** Naïve (CD4+/CD44low/int) and memory (CD4+/CD44 high) CD4 T cells were isolated from 4 young and 5 aged mice by FACS, and global gene expression levels were measured using microarray. **b** The number of up- and down-regulated genes differentially expressed with an FDR ≤0.05, in both naïve and memory subsets. The number in middle represents number of overlapping genes between both subsets, and is a fraction of the total number reported for both naïve and memory groups (i.e., 121 genes up-regulated in naïve cells at FDR ≤0.05, 41 of which were also upregulated in memory cells). **c** The number of up- and down-regulated genes differentially expressed with an expanded FDR of ≤0.1, in both naïve and memory subsets. Analysis results for the expanded gene list are referred to in the results and complete data is available in the supplemental data

Naïve T cells up-regulate the surface protein CD44 indefinitely upon exposure to a cognate antigen, and thus high expression of CD44 is a well-established marker of memory cells [22–24]. We isolated splenocytes from young and aged mice, and used fluorescent activated cell sorting (FACS) to collect naïve (CD4^+^/CD44^low/intermediate^) and memory (CD4^+^/CD44^high^) cells from each animal (Additional file 1; Figure S1). We then purified total RNA from each sample and conducted microarray analysis using Illumina MouseWG-6 v2.0 Expression BeadChips (Fig. 1a). Using an initial false discovery rate (FDR) threshold of ≤ 0.05, we identified 185 unique genes that were differentially expressed between young and old naïve CD4^+^ cells, and 328 unique genes that were differentially expressed between young and old memory CD4^+^ cells (Fig. 1b, Additional file 2: Tables S1 and S2). Of these, 121 and 256 genes were up-regulated during aging in naïve and memory cells, respectively, 41 of which were up-regulated in both populations (Additional file 2: Tables S1 and S2). In turn, 64 and 98 genes were down-regulated during aging in naïve and memory cells, respectively, 9 of which were down-regulated in both populations (Fig. 1b, Additional file 2: Tables S1 and S2). In agreement with our previous results using this microarray technology on human CD4+ T cells [21], fold change in expression was generally modest, ranging from ≈ 1.2–2.8 fold in naïve cells and ≈ 1.1–8 fold in memory cells.

Because the number of genes passing significance cutoff at FDR ≤ 0.05 was relatively small compared to previous mouse results [17], and smaller gene lists sometimes limit the effectiveness of bioinformatic tools, we also created an expanded set of differentially expressed genes using a FDR threshold of ≤ 0.1 for supplemental use. Using this expanded FDR, we identified 548 unique genes that were differentially expressed between young and old naïve CD4^+^ cells, and 693 unique genes that were differentially expressed between young and old memory CD4^+^ cells (Fig. 1c, Additional file 2: Tables S1 and S2). Of these, 320 and 413 genes were up-regulated during aging in naïve and memory cells, respectively, 104 of which were up-regulated in both populations (Fig. 1c, Additional file 2: Tables S1 and S2). In turn, 228 and 280 genes were down-regulated during aging in naïve and memory cells, respectively, 38 of which were down-regulated in both populations (Fig. 1c, Additional file 2: Tables S1 and S2).

### Functional annotation of Age-genes

We first wanted to see if differentially expressed genes identified in naïve and memory cells would show enrichment for specific functional annotations and if so, whether these annotations would be shared or unique between the two subsets. To address these questions, we first performed singular enrichment analysis (using DAVID Bioinformatic Resources v6.7) for enriched Gene Ontology (GO) and functional terms (e.g., KEGG pathway) on our lists of genes that were up and down-regulated (FDR ≤ 0.05) in naïve and memory cells (Table 1). Among genes up-regulated with age in naïve cells, significant (FDR ≤ 0.05) enrichment was found for genes involved in immune response, cytokine-cytokine receptor interaction, and regulation of apoptosis (6 classified as positive and 4 as negative regulators of apoptosis per GO annotation). Genes up-regulated in memory T cells were also enriched for cytokine-cytokine receptor interaction (four of these, *Ccl5, Cxcr5, Tnfrs4,* and *Bmp7*, were also part of this term for naïve cells), as well as endopeptidase inhibitor activity, regulation of interleukin-10 production, positive regulation of immune system processes, and lymphocyte and B cell activation. Enrichment from our expanded gene list (FDR ≤ 0.1) provided similar results (Additional file 3). No significant enrichment was found among genes down-regulated during aging in naïve or memory subsets, although in the expanded gene list (FDR ≤ 0.1) down-regulated genes in naïve cells were enriched for RNA polymerase II transcription factor activity and transcriptional activator activity (Additional file 4). These findings indicate that gene expression changes that occur during aging of naïve and memory CD4^+^ T cells reflect specific functional programs that are both shared and distinct between the two subsets.

**Table 1:** Singular Enrichment Analysis results from DAVID v6.7

Gene list	Category	Term	Count	*P* Value	FDR
Naïve Up	GOTERM_BP_FAT	GO:0006955 ~ immune response	13	1.72E-05	0.000266
	GOTERM_CC_FAT	GO:0005576 ~ extracellular region	15	4.64E-05	0.000517
	KEGG_PATHWAY	mmu04060:Cytokine-cytokine receptor interaction	8	1.41E-04	0.001418
	GOTERM_BP_FAT	GO:0042981 ~ regulation of apoptosis	13	3.17E-04	0.004887
Naïve Down	N/A	N/A	N/A	N/A	
Memory Up	KEGG_PATHWAY	mmu04060:Cytokine-cytokine receptor interaction	12	1.34E-05	0.000151
	GOTERM_MF_FAT	GO:0004866 ~ endopeptidase inhibitor activity	7	9.61E-05	0.001289
	INTERPRO	IPR007110:Immunoglobulin-like	13	2.07E-04	0.002886
	GOTERM_CC_FAT	GO:0016021 ~ integral to membrane	58	3.68E-04	0.004519
	GOTERM_BP_FAT	GO:0032653 ~ regulation of interleukin-10 production	4	6.05E-04	0.00977
	GOTERM_BP_FAT	GO:0002684 ~ positive regulation of immune system process	11	8.28E-04	0.01335
	GOTERM_BP_FAT	GO:0051249 ~ regulation of lymphocyte activation	9	0.001606	0.025729
	GOTERM_BP_FAT	GO:0050871 ~ positive regulation of B cell activation	5	0.00307	0.048641
Memory Down	N/A	N/A	N/A	N/A	N/A

Lists of genes differentially expressed between young and old mice at FDR ≤0.05 in naïve and memory CD4+ T cells were used as input, with all expressed genes in naïve and memory cells used as background. Broad terms such as “signal” and “disulfide bond” were excluded. A FDR of 0.05 was used as a threshold for enriched terms. No terms were significantly enriched in down-regulated gene lists

To enhance our understanding of the functional consequences of age-related gene expression in naïve and memory CD4^+^ T cells, we next performed Gene Set Enrichment Analysis (GSEA) on our samples from both subsets (Table 2). Rather than classifying genes based on pre-defined statistical cutoffs, GSEA is performed on all of the detectable probes measured within each sample, and ranks how well genes correlate with each phenotype (e.g., young and old), and then looks for over-representation of genes from predefined categories (e.g., oxidative phosphorylation) near the top or bottom of these lists. The major advantage of this method is that it can identify functional groups comprised of many genes with small fold-changes in the same direction that would otherwise be excluded by traditional statistical cutoffs. Gene transcripts down-regulated with age in naïve cells were enriched for Myc target genes, oxidative phosphorylation, DNA repair, epigenetic regulation of gene expression, and ribonucleoprotein complex. Genes up-regulated with age in naïve cells were enriched for a variety of different functions. Many gene sets up-regulated in naïve cells were involved in specific cytokine signaling pathways, including genes involved in TNFα signaling via NF-κB and genes activated by STAT5 in response to IL-2 signaling. GSEA also reiterated the increase in apoptosis genes in aged naïve T cells, although this was also accompanied by an increase in pro-proliferative genes. Memory cells showed similar enrichment of many of the same gene sets as naïve cells, with oxidative phosphorylation and Myc target genes being down-regulated with age, and genes involved in TNFα signaling via NF-κB, IL-2/STAT5 signaling, and IFNγ response (*Ifng* gene expression itself was up-regulated in aged memory cells, but not in naïve) being up-regulated with age. Together these results suggest that down-regulation of oxidative phosphorylation and MYC target genes and up-regulation of particular cytokine signaling pathways during aging is shared by epigenetic regulator and ribonucleoprotein complex genes, and age-related increase in apoptotic and cell proliferation genes.

**Table 2:** Gene Set Enrichment Analysis results

Enriched in Naïve Young (vs. Naïve Old) *Hallmarks*	SIZE	FDR	Enriched in Memory Young (vs. Memory Old) *Hallmarks*	SIZE	FDR
MYC_TARGETS_V1	155	0.001215	OXIDATIVE_PHOSPHORYLATION	163	0.001571
MYC_TARGETS_V2	40	0.002431	G2M_CHECKPOINT	128	0.011848
OXIDATIVE_PHOSPHORYLATION	162	0.002438	E2F_TARGETS	144	0.017543
DNA_REPAIR^a^	119	0.056895	MYC_TARGETS_V1	157	0.022091
			ANDROGEN_RESPONSE	64	0.02355
Enriched in Naïve Young (vs. Naïve Old)	SIZE	FDR	Enriched in Memory Young (vs. Memory Young) *GO terms*	SIZE	FDR
REGULATION_OF_GENE_EXPRESSION_EPIGENETIC	19	0.040616	N/A	N/A	N/A
RIBONUCLEOPROTEIN_COMPLEX^a^	95	0.057685			
Enriched in Naïve Old (vs. Naïve Young)	SIZE	FDR	Enriched in Memory Old (vs. Memory Young) *Hallmarks*	SIZE	FDR
IL2_STAT5_SIGNALING	128	0	TNFA_SIGNALING_VIA_NFKB	118	0.00186
TNFA_SIGNALING_VIA_NFKB	112	0	IL2_STAT5_SIGNALING	142	0.002496
INTERFERON_GAMMA_RESPONSE	130	0	INTERFERON_GAMMA_RESPONSE	135	0.010827
COMPLEMENT	92	2.19E-04	KRAS_SIGNALING_UP	80	0.029101
IL6_JAK_STAT3_SIGNALING	44	2.74E-04	IL6_JAK_STAT3_SIGNALING	52	0.035991
INFLAMMATORY_RESPONSE	92	7.16E-04	ALLOGRAFT_REJECTION	129	0.039874
KRAS_SIGNALING_UP	79	7.53E-04	INFLAMMATORY_RESPONSE	98	0.046285
ALLOGRAFT_REJECTION	126	0.001159			
APOPTOSIS	100	0.012965			
ESTROGEN_RESPONSE_EARLY	80	0.015578			
EPITHELIAL_MESENCHYMAL_TRANSITION	53	0.030826			
CHOLESTEROL_HOMEOSTASIS	40	0.045692			
Enriched in Naïve Old (vs. Naïve Young)	SIZE	FDR	Enriched in Memory Old (vs. Memory Young) *GO Terms*	SIZE	FDR
IMMUNE_RESPONSE	122	0.002943	N/A	N/A	N/A
CYTOKINE_ACTIVITY	26	0.014125			
CYTOKINE_BINDING	25	0.014879			
VIRAL_REPRODUCTIVE_PROCESS	22	0.045368			
PROTEIN_TYROSINE_KINASE_ACTIVITY	21	0.045902			
TRANSMEMBRANE_RECEPTOR_ACTIVITY	70	0.046233			
CYSTEINE_TYPE_ENDOPEPTIDASE_ACTIVITY	24	0.051925			
POSITIVE_REGULATION_OF_CELL_PROLIFERATION^a^	59	0.055409			

Association of a term with phenotype (i.e., Young or Old) indicates genes which comprise that term are more highly expressed within the phenotype (i.e., MYC_TARGETS_V1 are more highly expressed in young naive cells than in old naïve cells). Size indicates number of genes from each term that were enriched within phenotype. “Hallmarks” and “GO terms” indicate gene sets database used for analysis

^a^indicates terms that were slightly above significance cutoff (FDR 0.05) but were included in results because of previously established relevance to T cell aging

### Cis-regulators of naïve and memory age-genes

To determine potential upstream regulators of genes whose expression was altered by age, we next utilized i-cisTarget [25], a web tool which allows analysis of the regulatory regions of gene lists for enrichment of transcription factor binding sites (TFBSs; consensus DNA sequence to which a transcription factor binds; cataloged as position weight matrices [PWM]), previously mapped ChIP-seq results, and previously mapped histone modifications (Table 3). In naïve cells, genes up-regulated with age were most enriched for ChIP-seq peaks from the CHD1 transcription factor, although this dataset was from experiments performed in the CH12 (mouse B-cell lymphoma) cell line. The next top hits were all derived from archived consensus TFBSs, and indicate enrichment for NFκB/Rel, Runx family, and Gapb1 (Pu.1) TFBSs near genes up-regulated with age in naïve cells. Interestingly, the top 3 enriched features of genes down-regulated with age in naïve T cells all dealt with histone acetylation, particular in regards to epigenetic enhancer function. The top feature was H3K27ac (a mark of active enhancers [26]) ChIP-seq peaks from mouse thymus and the third ranked feature was H3K4me1 (a general mark of poised or active enhancers) ChIP-seq peaks from mouse thymus. The second ranked feature was ChIP-seq peaks for the histone acetyltransferase EP300, which also indicates enhancer regions [27], although these ChIP-seq peaks were collected from mouse heart tissue and thus the relevance to T cells is more uncertain. The sole nucleotide motif which passed our enrichment threshold (see methods) for genes down-regulated with age in naïve cells was a TFBS for several class O forkhead box (Foxo) transcription factors. In memory T cells, genes that changed with age were associated with fewer features. Up-regulated genes were only enriched for a Foxd3 TFBS, and down-regulated genes did not show any enriched features. Similar results were achieved with our expanded FDR (FDR ≤ 0.1) gene list (Additional file 5), although NF-κB became the top feature for genes up-regulated in memory cells and Foxo and Foxd3 TFBSs were no longer enriched near genes up-regulated in naïve and down-regulated in memory cells, respectively.

**Table 3:**
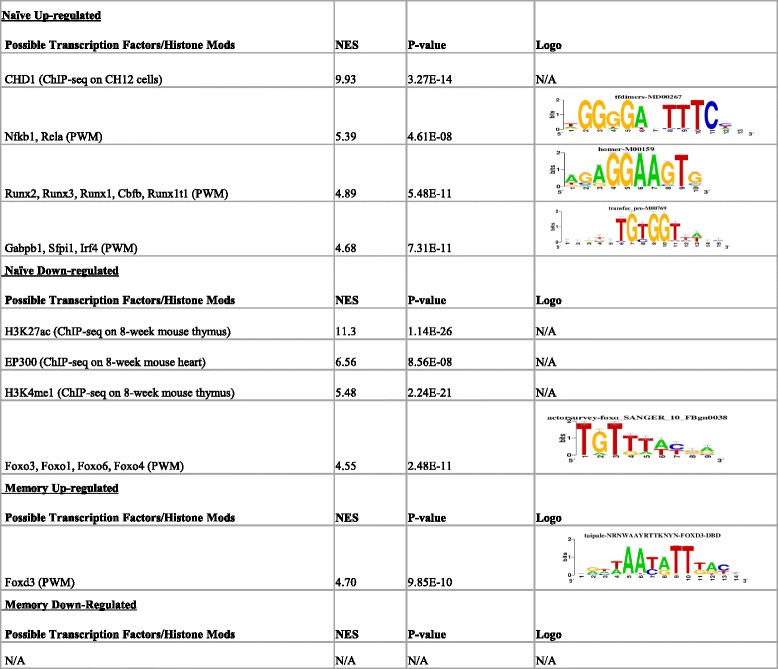
Cis-regulatory analysis of genes differentially expressed (FDR ≤0.05) during aging by i-cisTarget

Table shows transcription factor binding sites and histone modifications found to be enriched in +/− 10 kb regions flanking transcription start site of target genes (excluding coding regions). Parenthesis indicate database from which enriched feature was derived. NES = Normalized Enrichment Score. PWM = Positional Weight Matrix. *P*-value calculated using hypergeometric test

We also used the webtool oPOSSUM3 [28], which conducts similar analyses of gene lists based on PWM scoring (see methods for description), to analyze the cis-regulator regions of genes differentially expressed with age (Table 4). For genes up-regulated in naïve cells, no specific TFBS passed our enrichment threshold (Z-score ≥ 7, Fisher p-value ≤ 0.01; see methods); however, NF-κB was the top ranked TFBS by Fisher score and had a Z-score (Z-score = 6.6) just below our cutoff. Thus two independent methods identified enrichment of NF-κB binding sites in the regulatory regions of genes up-regulated during aging in naïve T cells. Genes down-regulated in naïve cells showed enrichment for a number of TFBSs, including interleukin responsive transcription factors Nfil3 and Gfi, and forkhead box transcription factors Foxo3 (also identified by i-cisTarget), FoxI1, and Foxq1. In memory cells, the binding site for IRF-1 (Interferon regulatory factor 1) was highly enriched amongst up-regulated genes as a function of both z-score and Fisher score (Z-score = 15.9; Fisher *p*-value = 4.7E-08). IRF-1 functions both as a tumor suppressor and regulator of immune response, and is required for IFNγ-mediated T_H_1 differentiation in CD4^+^ T cells [29]. Although they fell slightly below our Z or F-score cutoffs, we also found marginal enrichment for NF-κB, Foxq1, and Foxd3 (the top TFBS for up-regulated memory genes identified by i-cisTarget) binding sites near genes up-regulated in memory cells. Like naïve cells, genes down-regulated in memory cells were enriched for a larger variety of TFBSs than up-regulated genes, including several that were also enriched near genes down-regulated in naïve cells (Gfi, Nfil3, ELF5, and Prrx2). Similar results were achieved with our expanded FDR (FDR ≤ 0.1) gene list (Additional file 6) although, as with i-cisTarget results, NF-κB TFBSs became more enriched near up-regulated memory genes and Foxo and Foxd3 TFBSs were no longer enriched near naïve up and memory down-regulated genes, respectively.

**Table 4:** Cis-regulatory analysis of genes differentially expressed (FDR ≤0.05) during aging by oPOSSUM-3

Cell Type	Direction	TFBS	# of Targets	Z-score	F *p*-value	Cell Type	Direction	TFBS	# of Targets	Z-score	F *p*-value
Naïve	Up	NF-kappaB*	56	6.6	0.00197	Memory	Up	IRF1	82	15.916	4.7098E-08
Naïve	Up	Ar*	6	10.673	0.01202	Memory	Up	HOXA5	170	15.025	0.00065615
						Memory	Up	Ar	12	13.167	0.00010069
						Memory	Up	HNF1B	42	11.353	0.00990832
						Memory	Up	PPARG::RXRA	72	7.257	0.00070307
						Memory	Up	Gata1	152	6.963	0.00028379
						Memory	Up	NFKB1*	45	6.94	0.00058749
						Memory	Up	Foxq1*	92	6.759	6.2087E-05
						Memory	Up	Foxd3*	113	9.875	0.08090959
Cell Type	Direction	TFBS	# of Targets	Z-score	F *p*-value	Cell Type	Direction	TFBS	# of Targets	Z-score	F *p*-value
Naïve	Down	Prrx2	46	19.911	0.00112	Memory	Down	TP53	1	12.016	0.0043451
Naïve	Down	FOXI1	39	16.131	0.004315	Memory	Down	Gfi	64	11.621	0.00210378
Naïve	Down	Nobox	43	15.018	0.002301	Memory	Down	Ar	7	9.839	6.9823E-05
Naïve	Down	NFIL3	23	14.324	0.009441	Memory	Down	TEAD1	38	9.762	3.2211E-09
Naïve	Down	Gfi	45	13.979	0.000217	Memory	Down	TBP	59	9.326	4.8641E-08
Naïve	Down	Sox17	45	13.953	0.002388	Memory	Down	FEV	73	9.251	7.0307E-06
Naïve	Down	FOXO3	44	13.693	0.006281	Memory	Down	NFIL3	37	9.063	0.00019231
Naïve	Down	Foxq1	27	13.092	0.007568	Memory	Down	Lhx3	28	8.971	0.00107152
Naïve	Down	HLF	23	12.977	0.000107	Memory	Down	Pdx1	69	8.883	0.00365595
Naïve	Down	Gata1	47	12.834	0.000198	Memory	Down	FOXF2	25	8.74	8.3946E-05
Naïve	Down	Tal1::Gata1	24	12.074	0.001469	Memory	Down	NR3C1	19	8.273	7.9799E-06
Naïve	Down	CEBPA	41	10.428	0.004955	Memory	Down	Stat3	53	7.515	9.2897E-05
Naïve	Down	TBP	35	9.593	0.004688	Memory	Down	SPI1	74	7.368	1.3183E-05
Naïve	Down	SOX9	40	9.21	0.001014	Memory	Down	ELF5	77	7.248	4.14E-08
Naïve	Down	Myb	44	9.044	0.002547	Memory	Down	Egr1	37	7.203	7.4131E-06
Naïve	Down	RREB1	7	7.524	0.004571	Memory	Down	Prrx2	69	7.103	9.1411E-05
Naïve	Down	ELF5	51	7.382	3.47E-05						

Table shows transcription factor binding sites found to be enriched in +/− 10 kb regions flanking transcription start site of target genes (excluding coding regions). See Methods for explanation of Z-score and Fisher *p*-value

In summary, our cis-regulatory analyses suggest that altered activity and/or expression of several transcription factors and histone modifications may underlie the altered expression of these genes. In particular, our results point to increased NF-κB activity as a cause of increased gene expression in naïve and memory cells, a finding supported by our GSEA enrichment results (Table 2). Both analysis programs also identified Foxd3 binding sites near genes up-regulated in memory cells and Foxo binding sites near genes down-regulated in naïve cells. Finally, we identified enrichment of enhancer marks H3K27ac and H3K4me1 from thymus in the regulatory regions of genes down-regulated with age in naïve cells. Although enhancer marks are highly tissue specific [27], the fact that these marks were mapped in thymic tissue suggests a genuine involvement in T cell gene-regulation.

### Comparison to previous mouse and human results

A previous microarray study identified over 2,000 genes that were differentially expressed in naïve CD4^+^ T cells from young and aged mice [17] and we recently reported 186 genes with age-associated expression in human CD4^+^ T cells (FDR ≤0.01) [21]. We wanted to see which of the genes identified in the current study overlapped with previous results in mice and humans, in order to identify high confidence genes for future study-particularly those with relevance to human aging which can be studied in mouse models. Genes that were up- or down-regulated in more than one study are listed in in Table 5 (FDR ≤ 0.05) and Additional file 6 (FDR ≤ 0.1). Because CD4^+^ cells used in our human study contained a mixture of naïve and memory cells, we separately compared human results (converted to mouse orthologues, see Methods) to genes differentially expressed in naïve and memory CD4^+^ cells from the current study. We also calculated the probability of the observed vs. expected level of overlap in each comparison. In general, up-regulated genes showed more significant (hypergeometric test) overlap between datasets, especially between genes up-regulated in naïve cells from both mouse studies (p < 1.866e-15). The pro-inflammatory enzyme *Casp1* (Caspase-1) gene was up-regulated with age in all four datasets, and *Il1r2* (type 2 IL-1 receptor) was up-regulated in human CD4^+^ cells as well as both naïve and memory cells from our current experiments. Interestingly, *Casp1* and *Il1r2* have biologically antagonistic roles, with Casp1 protein functioning to cleave pro-IL-1β into the mature pro-inflammatory cytokine IL-1β, and IL-1r2 acting as a soluble decoy receptor that sequesters pro-IL-1β [30]. Also noteworthy is the up-regulation of *Dusp6* (dual specific phosphatase 6) transcripts in both of our naïve and memory groups along with our previous human results. Dusp6 is a phosphatase that was previously reported to increase with age in naïve human CD4^+^ T cells and cause defects in TCR signaling [14]. While there was little consistency amongst datasets for down-regulated genes, *Foxp1* (Forkhead box protein P1) was down-regulated with age in humans as well as both naive mouse CD4^+^ datasets. Foxp1 promotes T cell quiescence via suppression of both the *Il7r* gene and Erk signaling [31, 32], and is thought to antagonize the actions of Foxo1-which was also down-regulated at the transcript level in our human and naïve mouse datasets. Our comparison highlights several genes which change with age in T cells from both mice and humans, in particular up-regulation of *Casp1* and down-regulation of *Foxp1*.

**Table 5:** Comparison with previous mouse and human results

Dataset 1	Dataset 2	Direction	Genes	P-value
Human CD4+ *(118)*	Mouse Naïve *(119)*	*Up*	***Casp1*** *, Dusp6, Rgs1,*	*p < 0.021*
Human CD4+ *(118)*	Mouse Naïve (Mirza et al., 2011) *(734)*	*Up*	*Acvr2a, Aqp9, Arrdc4, Bcl2l2,* ***Casp1*** *, Cd86, Fcgr2b, Fgl2, Fndc3b,* ***Il1r2*** *, Ltb4r1, Rgs12, Lyn*	*p < 6.225e-05*
Human CD4+ *(118)*	Mouse Memory *(237)*	*Up*	*Bcl6,* ***Casp1*** *, Cyp4v3, Dusp6,* ***Il1r2*** *, Plcb2,*	*p < 0.001*
Mouse Naïve *(119)*	Mouse Naïve (Mirza et al., 2011) (734)	*Up*	*Abhd4, Adssl1, Bhlhb2, Bmp7,* ***Casp1*** *, Casp4, Xcl1, Ccl5, Cxcr3, Csprs, Endod1, Esm1, Eomes, Gbp3, Hmgn3, Ier3, Klf9, Lrrk1, Lpxn, Myo1f, Nkg7, Naip2, Ryk, Serpina9, Serpina3g, Zcchc18*	*p < 1.866e-15*
Human CD4+ *(181)*	Mouse Naïve (65)	*Down*	***Foxp1***	*p < 0.387*
Human CD4+ *(181)*	Mouse Naïve (Mirza et al., 2011) *(1382)*	*Down*	*Calb2, Epb4.1, Eef1d,* ***Foxp1*** *, Gpa33, Tgfbr2*	*p < 0.102*
Human CD4+ *(181)*	Mouse Memory *(107)*	*Down*	*l7Rn6, Trat1, Ube2n,*	*p < 0.047*
Mouse Naïve (65)	Mouse Naïve (Mirza et al., 2011) *(1382)*	*Down*	*Actn2, Actn1, Bcl9l, Ecm1,* ***Foxp1*** *, Mid1,*	*p < 0.166*

Rows performing comparison with data from current study include genes differentially expressed at FDR ≤0.05. Bolded terms were identified in multiple comparisons. Parenthesis indicate total number of genes used for comparison. Note that genes beginning with LOC were removed from gene lists from the current study for these comparisons, as they had been removed from the other studies, and thus the totals are slightly lower than reported in Fig. 1. P-values were calculated using the hypergeometric test

## Discussion

The age-related changes in functional networks we observed in both naïve and memory subsets echo previous reported as hallmarks of aging in the immune system and beyond. “Inflamm-aging” is a term used to define the well-established increase in low-grade inflammatory signaling associated with aging [33]. The increased expression of Il-2/Stat5 signaling, NF-κB target genes, IFNγ response, and inflammatory response genes with age seen in our GSEA results appear to reflect the effects of inflamm-aging. We find increased expression of cytokine and cytokine receptor genes in both naïve and memory subsets, a result previously observed in mouse and human T cells [16, 34]. Down-regulation of oxidative phosphorylation genes, which we observed in both subsets, is a common age-related phenotype for many tissues [21, 35]. In addition to increased “TNFα signaling via NF-κB” in aged cells from both subsets as indicated by GSEA, we also observed enrichment for putative NF-κB binding sites in the regulatory regions of genes up-regulated with age in both naïve and memory subsets (although this appeared to be stronger in naïve cells). Increased NF-κB signaling is another reoccurring feature of aging in the immune system [36] and other tissues [37], and has been proposed as a central cause of inflamm-aging [36]. Supporting the relevance of our findings in humans, a recent microarray study of aging human CD4^+^ T cells found gene expression mediated by NF-κB was increased during aging [20]. This same group also found aberrant activation of NF-κB target genes in the absence of stimulation in CD4+ T cells collected from older individuals [38]. In addition, our recently published work found that aging was associated with increased expression of the *NFKB1* gene in human CD4^+^ T cells [21]. Our findings support a major role of NF-κB in driving age-related gene expression changes in T cells.

One of the main distinguishing features we found for naïve CD4^+^ cells was an increase in genes with a role in apoptosis. The role of apoptosis in aging T cells appears complex; some studies report an age-related increase in apoptosis [6, 39] while others report an age-related impairment of apoptosis [7, 40]. The conflicting results may reflect differences in mice and humans. In mice, aging of naïve CD4^+^ cells is reportedly associated with longer cellular lifespan and reduced expression of the pro-apoptotic protein Bim [7]. It has been proposed that impaired apoptosis and increased lifespan leads to the accumulation of molecular damage and functional impairment in aged cells [7, 40]. Importantly, the genes we identified with an annotated role in apoptosis appear to have both pro-and anti-apoptotic roles, indicating a complex regulation of this cellular function in aged naïve cells. We also find that the pro-apoptotic *Casp1* gene is up-regulated with age in mice in both naïve and memory CD4^+^ cells, and also in human CD4^+^ cells.

Although it was just above our significance threshold (FDR = 0.058), we report that GSEA indicated a decline in ribonucleoprotein (RNP) complex genes in naïve CD4^+^ cells. The decline in RNP genes was also observed during aging in our recent study of human monocytes and CD4^+^ T cells [21], and was previously observed in aging human leukocytes [41]. Thus, decline in RNP genes may be a hallmark of T cell aging in mice and humans. Furthermore, RNP genes are regulated by Myc [42], and we also our GSEA results also indicate a decrease in Myc target genes. Thus, the decreased expression of RNP genes with age may be due to a dysfunctional or purposeful (i.e., compensatory) decline in Myc signaling. This is especially interesting considering the recent finding that decreased expression of Myc enhances lifespan and healthspan in mice [43].

We also observed specific enrichment of enhancer marks H3K27ac and H3K4me1 (derived from 8-week old mouse thymus) near genes down-regulated in naïve cells. Although enhancers are known to play a major role in gene regulation, little is currently known about the role of enhancers in aging. A recent study of human monocytes by our lab found that age-associated DNA methylation alterations which were also associated with cis-gene expression (age-eMS), were enriched for enhancer regions, indicated by H3K27ac and H3K4me1 marks (previously mapped in a young monocyte sample by ENCODE) [44]. Similarly, Shah et al. [45] found that H3K27me3 peaks lost during aging were strongly enriched for H3K27ac sites previously mapped in young fibroblasts. Overall, these results suggest that altered enhancer activity during aging may contribute to age-related changes in gene expression in naïve CD4^+^ T cells. Further studies are necessary to confirm specific regulatory functions identified in the current study - e.g., ChIP-seq of enhancer histone modification in young and aged T cells-before they are pursued as potential means of therapy to reverse age-related gene expression changes in T cells. As epigenetic modifications are highly cell-type specific, future studies may also benefit from further subset purification, e.g., central vs. effector memory populations.

Class O forkhead box (Foxo) transcription factors are well known regulators of both T cell function [46] and the aging process [47]. Our findings indicate that altered activity and/or expression of several different classes of forkhead box transcription factor genes, including Foxo1 and Foxo3, may be important in T cell aging. We find *Foxo1* mRNA is down-regulated in naïve mouse cells and human CD4 cells, together with the enrichment for putative Foxo binding sites near down-regulated genes in naïve cells. Foxo1 is a positive regulator of T cell proliferation via activation of the *Il7r* gene [31] (although we did not observe altered expression of *Il7r* in mice or humans) and thus decline in expression/activity may cause reduced proliferation of naïve cells with age. In support of reduced Foxo1 expression/activity with age in naïve T cells, we did observe decreased expression of *Foxo*1 target gene *Klf2* [46] but surprisingly Foxo1 target genes *Eomes* and *Ctla4* showed increased expression. Putative Foxd3 binding sites are enriched near genes up-regulated with age in memory cells. This gene does not have an established role in T cell biology but is thought to act as a tumor suppressor and regulator of pluripotency in embryonic stem cells [48]. However, *Foxd3* transcripts were not detectable in memory cells in our analysis. Although this does not preclude the possibility of *Foxd3* expression (transcription factor mRNAs are often expressed at very low levels), these results should be interpreted with caution. Finally, we find that the *Foxp1* gene is down-regulated during aging in naïve mouse CD4^+^ cells and human CD4^+^ cells. Foxp1 opposes Foxo1 function in T cells by repressing *Il7r* expression, thus promoting T cell quiescence [32]. The down-regulation of both *Foxo1* and *Foxp1* may be a result of compensatory mechanisms. Together these findings indicate potentially important subset specific roles for different Fox proteins in T cell aging.

Overlap between mouse results with our previous results from human CD4^+^ cells is low. This may be due to the heterogeneity of human CD4^+^ cells, or may reflect differences in mouse and human aging programs. However, our analysis did identify several interesting genes with similar age-related changes that occur consistently in mice and humans. Among these genes were *Casp1* and *Foxp1*, which we discuss above. We also found *Dusp6* to be up-regulated in naïve and memory CD4^+^ cells, as well as human CD4^+^ cells. Dusp6 expression was previously reported to increase in naïve CD4^+^ T cells during aging in humans and cause functional defects in TCR-ERK signaling [14], although this increase was reported at the protein but not mRNA level. Our findings indicate that *Dusp6* mRNA does with age, in both mouse (naïve and memory) and human CD4^+^ T cells. Murine models may therefore represent a clinically relevant model to study the cause and effect of age-related increase of Dusp6 in CD4^+^ cells and to test therapies to reverse this process.

One potential limitation of our study is the sole use of CD44 as a marker to distinguish naïve and memory T cells. The CD4+/CD44^high^ population is likely to be somewhat heterogeneous, containing both central and effector memory populations, as wells regulatory T cells. The use of additional markers, such as CD62 and FoxP3, in future studies would allow for isolation of more homogeneous populations. An additional limitation is the lack of validation studies-specifically qPCR to validate age-related changes in expression of key candidate genes identified by microarray, and ChIP-qPCR and/or ChIP-seq to validate potential age-related changes in binding of transcription factors and histone modifications in the regulatory regions of genes that show altered expression with age. Finally, we note that the microarray technology employed in this study is not as sensitive or comprehensive as RNA-seq.

## Conclusions

Our findings show that naïve and memory CD4^+^ subsets both undergo substantial changes in gene expression during the aging process. Genes with altered expression are generally distinct; however, functional annotation analyses indicate that aging affects a number of common gene expression networks in the two subsets. These common features include: increased expression of cytokine/cytokine receptor genes, decreased expression of oxidative phosphorylation and Myc target genes, up-regulation of NF-κB target genes, and increased inflammatory signaling. Our results also suggest that both subsets also exhibit unique transcriptomic alterations; specifically, genes up-regulated with age in naïve cells were specifically enriched for apoptotic signaling function, and genes down-regulated with age in naïve cells were enriched for nearby enhancer histone modifications and Foxo transcription factor binding sites. Memory cells, on the other hand, showed little specific enrichment for gene function; however, the regulator regions of age-associated genes in this subset did show enrichment of specific TFBSs near genes, particularly Foxd3 and Irf-1. Lastly, we show that several well-characterized genes previously reported to be affected by age in human CD4^+^ T cells (e.g., DUSP6) show similar expression changes in mice. However, an important finding from our comparison with human results was the low overall overlap of genes affected by age in humans and mice.

Both naïve and memory CD4+ T cells undergo age-related functional decline. Our study highlights specific genes and gene pathways that may underlie this functional decline. Furthermore, we also identify upstream regulatory factors that may potentially drive these changes and provide attractive targets for future studies on T cell aging. Finally, our findings highlight the need for caution when interpreting results from murine models of T cell aging.

### Acknowledgements

The MESA Epigenomics Study was funded by NHLBI grant R01HL101250 to Wake Forest University Health Sciences. The MESA Epigenomics & Transcriptomics Study was funded by NHLBI grant R01HL101250 to Wake Forest University Health Sciences. J.R.T. was supported by T32AG033534 from the National Institute of Aging.

## Methods

### Mice

C57BL/6 mice were used for all studies. Young mice (2–3 months) were acquired from Harlan Laboratories (Indianapolis, IN) and aged mice (28 months) were acquired from Charles River Laboratories (Wilmington, MA) via the National Institute on Aging (Bethesda, MD). Mice were sacrificed by cervical dislocation following anesthesia by isoflurane. Animal housing and procedures were approved by the Animal Care and Use Committee of Wake Forest University Health Sciences. Principles of laboratory animal care (NIH publication No. 86–23, revised 1985) were followed during euthanasia procedures.

### T cell isolation

For each mouse, the spleen was removed and homogenized in ice cold RPMI medium with 2% FBS. Cell suspensions were filtered through a 40 μM filter, and red blood cells were lysed with RBC Lysis buffer (Biolegend, San Diego, CA) per the manufacturer’s instructions. Cells were filtered again, then incubated with Fixable Viability Dye eFluor® 450 (eBioscience, San Diego, CA), a cocktail of FITC-conjugated lineage antibodies (CD11b, CD8a, CD49b, CD45RO, Gr-1, and Ter119), CD4-PE, and CD44-APC antibodies for 30 minutes at 4 °C, washed and resuspended in RPMI without phenol red ^+^ 2% FBS before proceeding FACS. All antibodies were purchased from eBiosciences (San Diego, CA) or BD Biosciences (San Jose, CA).

### FACS

FACS was performed on a FACS Aria cell sorter (BD Biosciences, San Jose, CA). Naïve (CD44^low/intermediate^) and memory (CD44^high^) Lin^−^/CD4^+^ T cells were sorted into cold FBS. After sorting, cells were pelleted at 500 × g for 10 min and lysed in QIAzol (QIAGEN, Valencia, CA) reagent before storage at −80 °C. Isotype and single color controls were used for every sort.

### RNA extraction

RNA extraction was performed using the miRNeasy Micro kit (QIAGEN, Valencia, CA) according to manufacturer directions. Eluted RNA was initially quantified using a Nanodrop. RNA quality and concentration was also assessed with a RNA 6000 Pico kit (Agilent Technologies, Santa Clara, CA) on a 2100 Bioanalyzer (Agilent Technologies). All samples had RIN values > 8.0, with an average value of 9.7.

### Global gene expression quantification

50 ng of total RNA from each sample was amplified and labeled using the Illumina® TotalPrep™-96 RNA Amplification Kit (Life Technologies, Carlsbad, CA). The MouseWG-6 v2.0 Expression BeadChip and Illumina Bead Array Reader were used to perform the genome-wide expression analysis, following the Illumina expression protocol. Seven hundred monogram of biotinylated cRNA was hybridized to a BeadChip at 58 °C for 16 – 17 h. To avoid potential biases due to batch, chip, and position effects, a stratified random sampling technique was used to assign individual samples to specific BeadChips (6 samples/chip) and chip position.

### Microarray data pre-processing and differential expression analysis

Background corrected bead-level data was obtained from Illumina GenomeStudio software and subsequent pre-processing, quality control, and statistical analysis were performed in *R* using *Bioconductor* packages. Quantile normalization was performed with the *neqc* function of the *limma* package, with the addition of a small recommended offset [49]. Normalized probe values were log_2_ transformed and control probe and outlier samples were eliminated from the expression matrix. Multidimensional scaling plots showed that naïve and memory samples clustered by cell type and age. One young naïve sample was removed due to low signal and one old memory sample was detected as an outlier by multidimensional scaling analysis and was also removed. Detection *p*-values were computed using negative controls from the beadarray. To detect differential expression between two groups with small sample sizes, the regularized *t*-test implemented in the limma R package was used [50]. The false discovery rate (FDR) using q-value method [51] was reported.

### Functional annotation analysis

Functional annotation of genes differentially expressed between young and old mice was performed using DAVID Bioinformatic Resources v6.7 [52]. Lists of up- and down-regulated Illumina probe IDs (FDR ≤ 0.05 for main text table and FDR ≤ 0.1 for supplemental table) were entered into the Functional Annotation Tool web application, with lists of all detectable Illumina probe IDs for naïve or memory cells used as background. An FDR ≤ 0.05 was used to define enrichment. Broad terms such as “signal” and “disulfide bond” were excluded from results. Gene Set Enrichment Analysis [53] was performed using the Java application available from The Broad Institute (www.broadinstitute.org/gsea/). As this software incorporates all (detectable) probe values for each sample, separate results for each FDR threshold were not necessary. Gene set databases used were Hallmarks (h.all.v5.0.symbols.gmt) and Gene Ontology (c5.all.v5.0.symbols.gmt). One thousand gene set permutations were performed. An FDR cutoff of ≤ 0.05 was used for enriched terms, as is recommended when performing permutations by gene set.

### Cis-regulatory analysis

i-cisTarget [25] and oPOSSUM-3 [28] web applications were used for analysis of cis-regulatory regions, defined in both programs as ^+^/- 10 kb from the transcription start site of each gene (excluding coding regions). For i-cisTarget, ROC threshold for AUC calculation was set to 0.005. i-cisTarget uses a ranking and recovery method which produces a Normalized Enrichment Score (NES). An NES of 3 roughly corresponds to a FDR ≤ 0.05 [54] and is the default enrichment cutoff for the program. However, we found NES scores up to 5.5 for ChIP-seq (although many reflected thymic tissue) and up to 4.3 for PWM (i.e., consensus DNA binding sequence for a transcription factor) database results when running lists of all detectable T cell genes, and thus used these scores as cutoffs for enrichment in order to compensate for tissue specific gene expression. For oPOSSUM-3, mouse Single Site Analysis was used with a conservation cutoff of 0.60 and a matrix score threshold of 85%. A list of all detectable genes in naïve or memory cells was used for background. oPOSSUM analyzes TFBS enrichment through both Z-score and F-score, which is the negative log of the Fisher one-tailed exact probability. The Z-score used compare the rate of occurrence of a TFBS in the target set of genes to the expected rate estimated from the pre-computed background. F-score is based on the one-tailed Fisher exact probability, which compares the proportion of co-expressed genes containing a particular TFBS to the proportion contained in the background set. Thus the F-score/*p*-value does not consider the number of times a TFBS appears near a gene beyond once. For enrichment of TFBSs, we employed a combined threshold of 7 for Z-score and 2 (corresponding to a *p*-value of 0.01) for F-score, based on available recommendations and literature [55].

### Comparison with previous datasets

For comparison of mouse and human gene lists, human genes were converted to mouse orthologs using the ENSMBL Biomart tool. Analyzed data from Mirza et al. [17] was downloaded from the NCBI Gene Expression Omnibus (Accession number GSE28165). A hypergeometric test was used to compare significance of overlap between two datasets. The test was performed using a publically available calculator (http://nemates.org/MA/progs/overlap_stats.html).

## Additional files

Additional file 1: Figure S1. FACS profile for sorting naïve and memory CD4+ T cells. Representative gates for FACS setup used to separate naïve and memory CD4+ T cells. Spleen cells were separated based on forward and side scatter (P1 and P2). Dead cells (violet positive) were removed (P3), as were lineage/FITC positive (see methods) (P4), before CD4+ cells (y-axis, bottom left plot) were separated based on CD44 expression (x-axis, bottom left plot) to isolate naïve (CD44 low) and memory (CD44 high) populations. Bottom right plot shows alternate representation of CD44 expression. (PDF 353 kb)

Additional file 2: Genes differentially expressed in young and old, memory and naïve CD4+ T cells. list of gene symbols, Illumina probe IDs, log2 fold change, *p*-values and FDR for all genes differentially expressed between young and old naïve (CD44 Low) and young and old memory (CD44 high) at a FDR of ≤ 0.1. (XLSX 125 kb)

Additional file 3: Table S1. DAVID results from expanded gene list (FDR ≤0.1). Lists of genes differentially expressed between young and old mice at FDR ≤0.1 in naïve and memory CD4+ T cells were used as input, with all expressed genes in naïve and memory cells used as background. Broad terms such as “signal” and “disulfide bond” were excluded. A FDR of 0.05 was used as a threshold for enriched terms. No terms were significantly enriched in down-regulated gene lists. (PPTX 40 kb)

Additional file 4: Table S2. Cis-regulatory analysis of expanded gene list (FDR ≤0.1) by i-cisTarget. Table shows transcription factor binding sites and histone modifications found to be enriched in +/− 10 kb regions flanking transcription start site of target genes (excluding coding regions). Parenthesis indicate database from which enriched feature was derived. NES = Normalized Enrichment Score. PWM = Positional Weight Matrix. *P*-value calculated using hypergeometric test. (PPTX 56 kb)

Additional file 5: Table S3. Cis-regulatory analysis of genes differentially expressed (FDR ≤0.1) during aging by oPOSSUM-3. Table shows transcription factor binding sites found to be enriched in +/− 10 kb regions flanking transcription start site of target genes (excluding coding regions). See Methods for explanation of Z-score and Fisher *p*-value. (PPTX 42 kb)

Additional file 6: Table S4. Comparison with previous mouse and human results. Rows performing comparison with data from current study include genes differentially expressed at FDR ≤0.1. * indicates value or gene from the current study differentially expressed at FDR < 0.05; those without a * were differentially expressed at a FDR >0.05 and ≤ 0.1. Bolded terms were identified in multiple comparisons. Parenthesis indicate total number of genes used for comparison. Note that genes beginning with LOC were removed from gene lists from the current study for these comparisons, as they had been removed from the other studies, and thus the totals are slightly lower than reported in Fig. 1. *P*-values were calculated using the hypergeometric test. (PPTX 44 kb)
