# Supplementary figures and images for: Transcriptomic profiles of aging in naïve and memory CD4+ cells from mice
Source: Immun Ageing. 2017 Jun 20;14:15. doi: 10.1186/s12979-017-0092-5 (PMC5477126; doi:10.1186/s12979-017-0092-5)

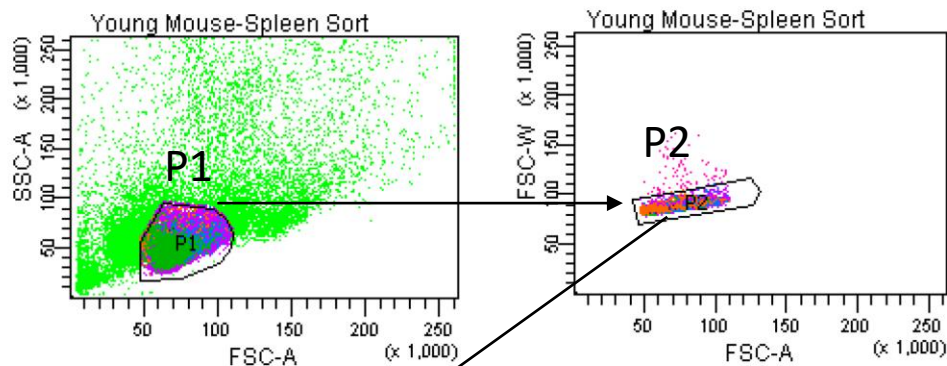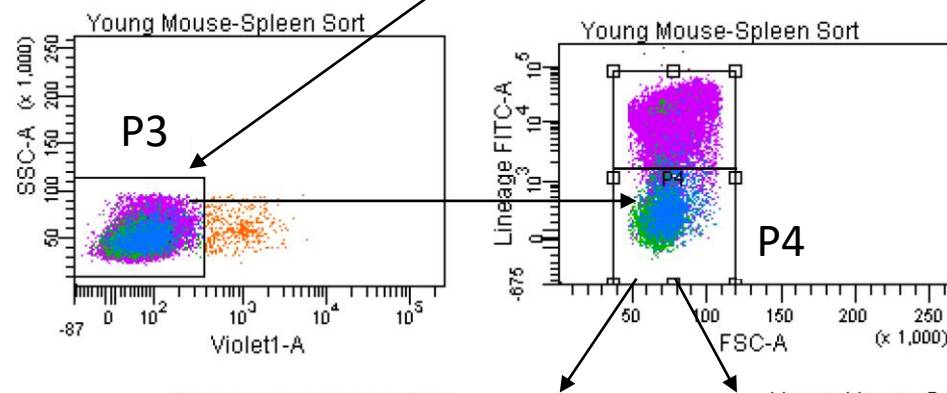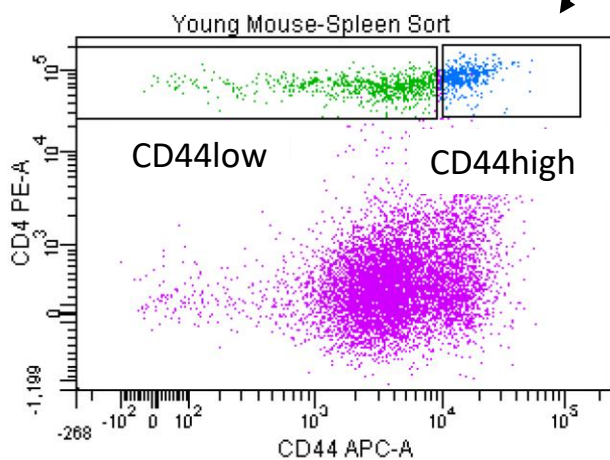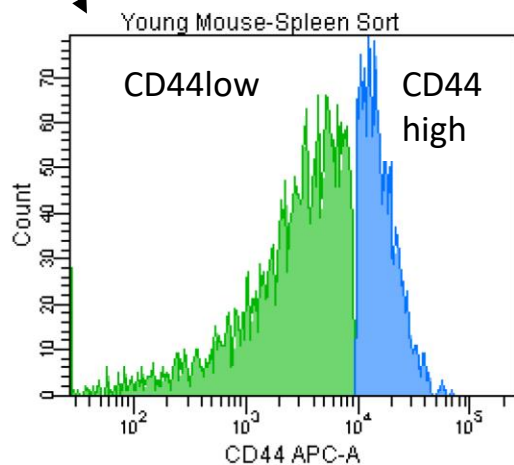

Supplement: Supplementary file 1 — FACS profile for sorting naïve and memory CD4+ T cells. Representative gates for FACS setup used to separate naïve and memory CD4+ T cells. Spleen cells were separated based on forward and side scatter (P1 and P2). Dead cells (violet positive) were removed (P3), as were lineage/FITC positive (see methods) (P4), before CD4+ cells (y-axis, bottom left plot) were separated based on CD44 expression (x-axis, bottom left plot) to isolate naïve (CD44 low) and memory (CD44 high) populations. Bottom right plot shows alternate representation of CD44 expression. (PDF 353 kb) [file 12979_2017_92_MOESM1_ESM.pdf]
