# Supplementary material for: Transcriptomic profiles of aging in naïve and memory CD4+ cells from mice
Source: Immun Ageing. 2017 Jun 20;14:15. doi: 10.1186/s12979-017-0092-5 (PMC5477126; doi:10.1186/s12979-017-0092-5)
Supplement: Supplementary file 3 — DAVID results from expanded gene list (FDR ≤0.1). Lists of genes differentially expressed between young and old mice at FDR ≤0.1 in naïve and memory CD4+ T cells were used as input, with all expressed genes in naïve and memory cells used as background. Broad terms such as “signal” and “disulfide bond” were excluded. A FDR of 0.05 was used as a threshold for enriched terms. No terms were significantly enriched in down-regulated gene lists. (PPTX 40 kb) [file 12979_2017_92_MOESM3_ESM.pptx]

## Slide 1
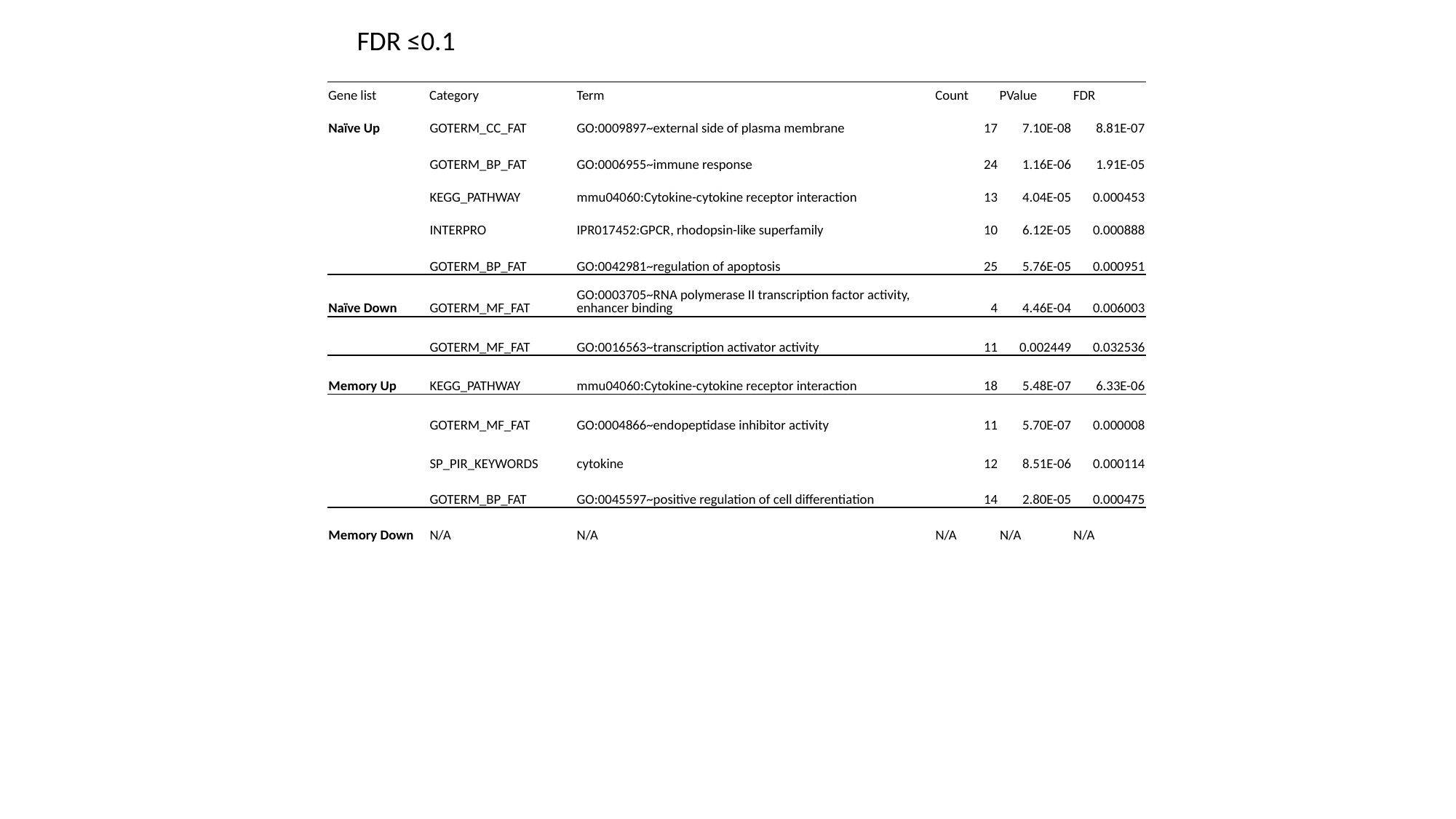

FDR ≤0.1
| Gene list | Category | Term | Count | PValue | FDR |
| --- | --- | --- | --- | --- | --- |
| Naïve Up | GOTERM\_CC\_FAT | GO:0009897~external side of plasma membrane | 17 | 7.10E-08 | 8.81E-07 |
| | GOTERM\_BP\_FAT | GO:0006955~immune response | 24 | 1.16E-06 | 1.91E-05 |
| | KEGG\_PATHWAY | mmu04060:Cytokine-cytokine receptor interaction | 13 | 4.04E-05 | 0.000453 |
| | INTERPRO | IPR017452:GPCR, rhodopsin-like superfamily | 10 | 6.12E-05 | 0.000888 |
| | GOTERM\_BP\_FAT | GO:0042981~regulation of apoptosis | 25 | 5.76E-05 | 0.000951 |
| Naïve Down | GOTERM\_MF\_FAT | GO:0003705~RNA polymerase II transcription factor activity, enhancer binding | 4 | 4.46E-04 | 0.006003 |
| | GOTERM\_MF\_FAT | GO:0016563~transcription activator activity | 11 | 0.002449 | 0.032536 |
| Memory Up | KEGG\_PATHWAY | mmu04060:Cytokine-cytokine receptor interaction | 18 | 5.48E-07 | 6.33E-06 |
| | GOTERM\_MF\_FAT | GO:0004866~endopeptidase inhibitor activity | 11 | 5.70E-07 | 0.000008 |
| | SP\_PIR\_KEYWORDS | cytokine | 12 | 8.51E-06 | 0.000114 |
| | GOTERM\_BP\_FAT | GO:0045597~positive regulation of cell differentiation | 14 | 2.80E-05 | 0.000475 |
| Memory Down | N/A | N/A | N/A | N/A | N/A |
