# Supplementary material for: Transcriptomic profiles of aging in naïve and memory CD4+ cells from mice
Source: Immun Ageing. 2017 Jun 20;14:15. doi: 10.1186/s12979-017-0092-5 (PMC5477126; doi:10.1186/s12979-017-0092-5)
Supplement: Supplementary file 4 — Cis-regulatory analysis of expanded gene list (FDR ≤0.1) by i-cisTarget. Table shows transcription factor binding sites and histone modifications found to be enriched in +/− 10 kb regions flanking transcription start site of target genes (excluding coding regions). Parenthesis indicate database from which enriched feature was derived. NES = Normalized Enrichment Score. PWM = Positional Weight Matrix. P-value calculated using hypergeometric test. (PPTX 56 kb) [file 12979_2017_92_MOESM4_ESM.pptx]

## Slide 1
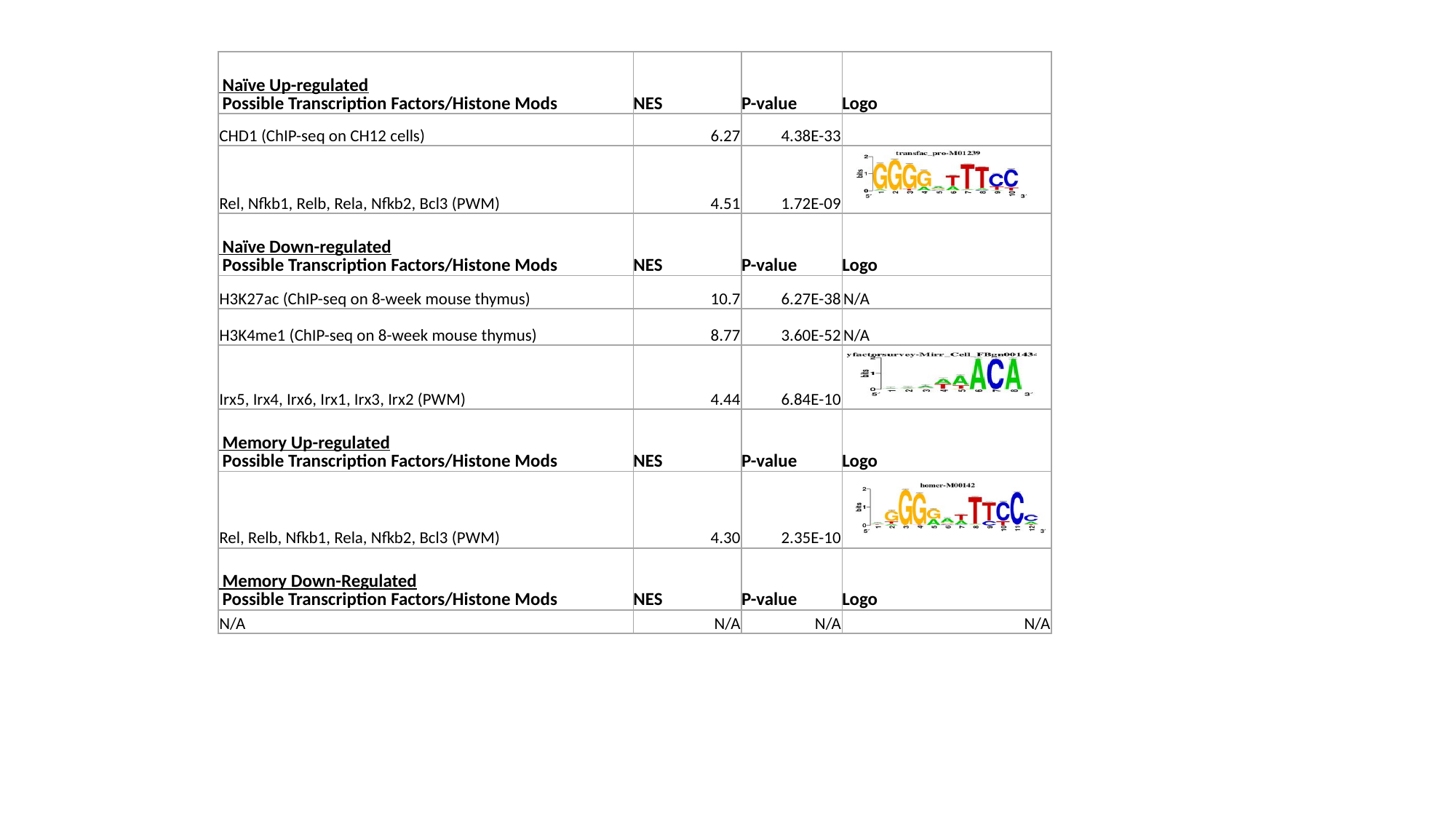

| Naïve Up-regulated Possible Transcription Factors/Histone Mods | NES | P-value | Logo |
| --- | --- | --- | --- |
| CHD1 (ChIP-seq on CH12 cells) | 6.27 | 4.38E-33 | |
| Rel, Nfkb1, Relb, Rela, Nfkb2, Bcl3 (PWM) | 4.51 | 1.72E-09 | |
| Naïve Down-regulated Possible Transcription Factors/Histone Mods | NES | P-value | Logo |
| H3K27ac (ChIP-seq on 8-week mouse thymus) | 10.7 | 6.27E-38 | N/A |
| H3K4me1 (ChIP-seq on 8-week mouse thymus) | 8.77 | 3.60E-52 | N/A |
| Irx5, Irx4, Irx6, Irx1, Irx3, Irx2 (PWM) | 4.44 | 6.84E-10 | |
| Memory Up-regulated Possible Transcription Factors/Histone Mods | NES | P-value | Logo |
| Rel, Relb, Nfkb1, Rela, Nfkb2, Bcl3 (PWM) | 4.30 | 2.35E-10 | |
| Memory Down-Regulated Possible Transcription Factors/Histone Mods | NES | P-value | Logo |
| N/A | N/A | N/A | N/A |
