# Supplementary material for: Transcriptomic profiles of aging in naïve and memory CD4+ cells from mice
Source: Immun Ageing. 2017 Jun 20;14:15. doi: 10.1186/s12979-017-0092-5 (PMC5477126; doi:10.1186/s12979-017-0092-5)
Supplement: Supplementary file 5 — Cis-regulatory analysis of genes differentially expressed (FDR ≤0.1) during aging by oPOSSUM-3. Table shows transcription factor binding sites found to be enriched in +/− 10 kb regions flanking transcription start site of target genes (excluding coding regions). See Methods for explanation of Z-score and Fisher p-value. (PPTX 42 kb) [file 12979_2017_92_MOESM5_ESM.pptx]

## Slide 1
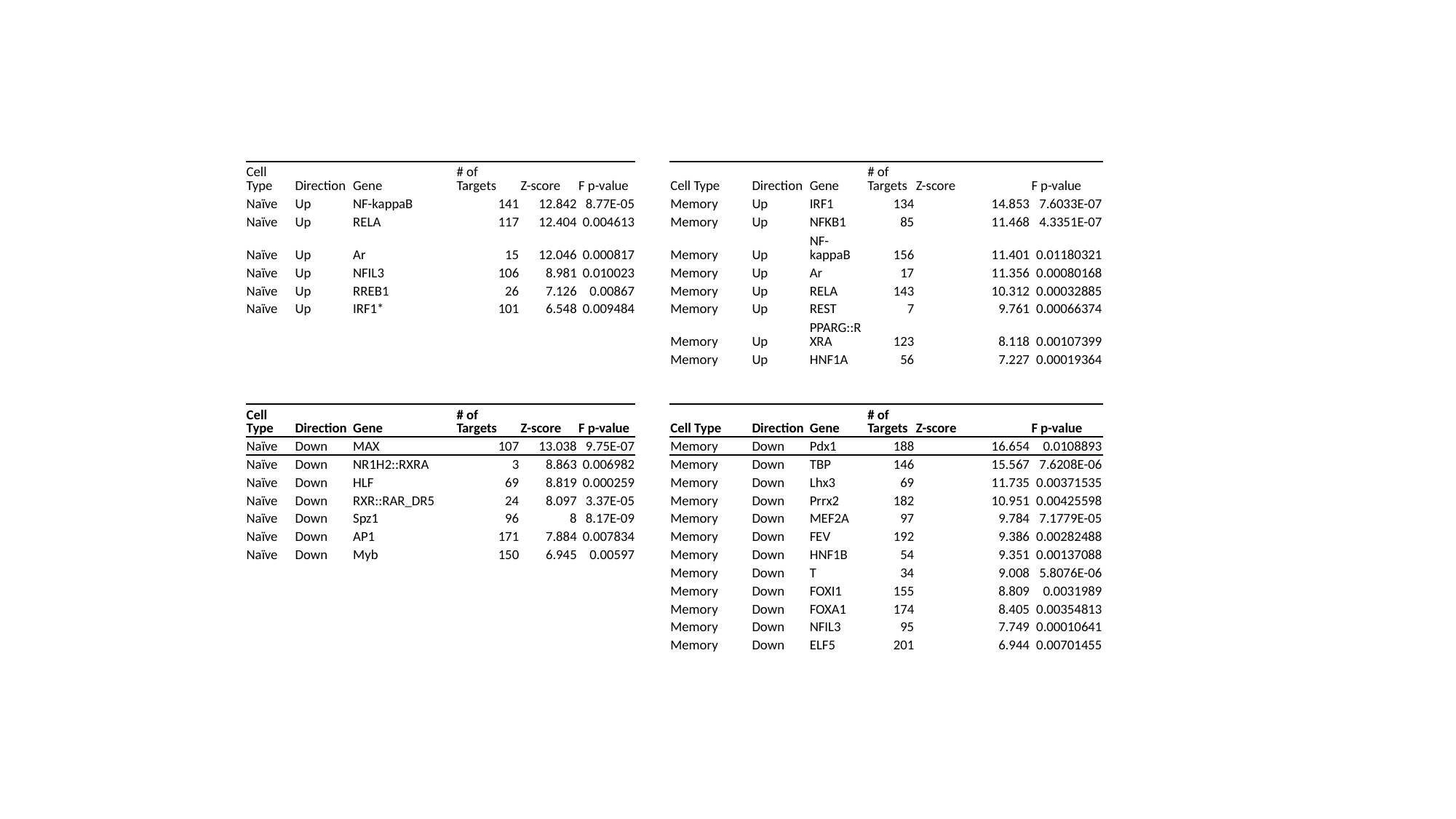

| Cell Type | Direction | Gene | # of Targets | Z-score | F p-value | | Cell Type | Direction | Gene | # of Targets | Z-score | F p-value |
| --- | --- | --- | --- | --- | --- | --- | --- | --- | --- | --- | --- | --- |
| Naïve | Up | NF-kappaB | 141 | 12.842 | 8.77E-05 | | Memory | Up | IRF1 | 134 | 14.853 | 7.6033E-07 |
| Naïve | Up | RELA | 117 | 12.404 | 0.004613 | | Memory | Up | NFKB1 | 85 | 11.468 | 4.3351E-07 |
| Naïve | Up | Ar | 15 | 12.046 | 0.000817 | | Memory | Up | NF-kappaB | 156 | 11.401 | 0.01180321 |
| Naïve | Up | NFIL3 | 106 | 8.981 | 0.010023 | | Memory | Up | Ar | 17 | 11.356 | 0.00080168 |
| Naïve | Up | RREB1 | 26 | 7.126 | 0.00867 | | Memory | Up | RELA | 143 | 10.312 | 0.00032885 |
| Naïve | Up | IRF1\* | 101 | 6.548 | 0.009484 | | Memory | Up | REST | 7 | 9.761 | 0.00066374 |
| | | | | | | | Memory | Up | PPARG::RXRA | 123 | 8.118 | 0.00107399 |
| | | | | | | | Memory | Up | HNF1A | 56 | 7.227 | 0.00019364 |
| | | | | | | | | | | | | |
| | | | | | | | | | | | | |
| Cell Type | Direction | Gene | # of Targets | Z-score | F p-value | | Cell Type | Direction | Gene | # of Targets | Z-score | F p-value |
| Naïve | Down | MAX | 107 | 13.038 | 9.75E-07 | | Memory | Down | Pdx1 | 188 | 16.654 | 0.0108893 |
| Naïve | Down | NR1H2::RXRA | 3 | 8.863 | 0.006982 | | Memory | Down | TBP | 146 | 15.567 | 7.6208E-06 |
| Naïve | Down | HLF | 69 | 8.819 | 0.000259 | | Memory | Down | Lhx3 | 69 | 11.735 | 0.00371535 |
| Naïve | Down | RXR::RAR\_DR5 | 24 | 8.097 | 3.37E-05 | | Memory | Down | Prrx2 | 182 | 10.951 | 0.00425598 |
| Naïve | Down | Spz1 | 96 | 8 | 8.17E-09 | | Memory | Down | MEF2A | 97 | 9.784 | 7.1779E-05 |
| Naïve | Down | AP1 | 171 | 7.884 | 0.007834 | | Memory | Down | FEV | 192 | 9.386 | 0.00282488 |
| Naïve | Down | Myb | 150 | 6.945 | 0.00597 | | Memory | Down | HNF1B | 54 | 9.351 | 0.00137088 |
| | | | | | | | Memory | Down | T | 34 | 9.008 | 5.8076E-06 |
| | | | | | | | Memory | Down | FOXI1 | 155 | 8.809 | 0.0031989 |
| | | | | | | | Memory | Down | FOXA1 | 174 | 8.405 | 0.00354813 |
| | | | | | | | Memory | Down | NFIL3 | 95 | 7.749 | 0.00010641 |
| | | | | | | | Memory | Down | ELF5 | 201 | 6.944 | 0.00701455 |
