# Supplementary material for: Transcriptomic profiles of aging in naïve and memory CD4+ cells from mice
Source: Immun Ageing. 2017 Jun 20;14:15. doi: 10.1186/s12979-017-0092-5 (PMC5477126; doi:10.1186/s12979-017-0092-5)
Supplement: Supplementary file 6 — Comparison with previous mouse and human results. Rows performing comparison with data from current study include genes differentially expressed at FDR ≤0.1. * indicates value or gene from the current study differentially expressed at FDR < 0.05; those without a * were differentially expressed at a FDR >0.05 and ≤ 0.1. Bolded terms were identified in multiple comparisons. Parenthesis indicate total number of genes used for comparison. Note that genes beginning with LOC were removed from gene lists from the current study for these comparisons, as they had been removed from the other studies, and thus the totals are slightly lower than reported in Fig. 1. P-values were calculated using the hypergeometric test. (PPTX 44 kb) [file 12979_2017_92_MOESM6_ESM.pptx]

## Slide 1
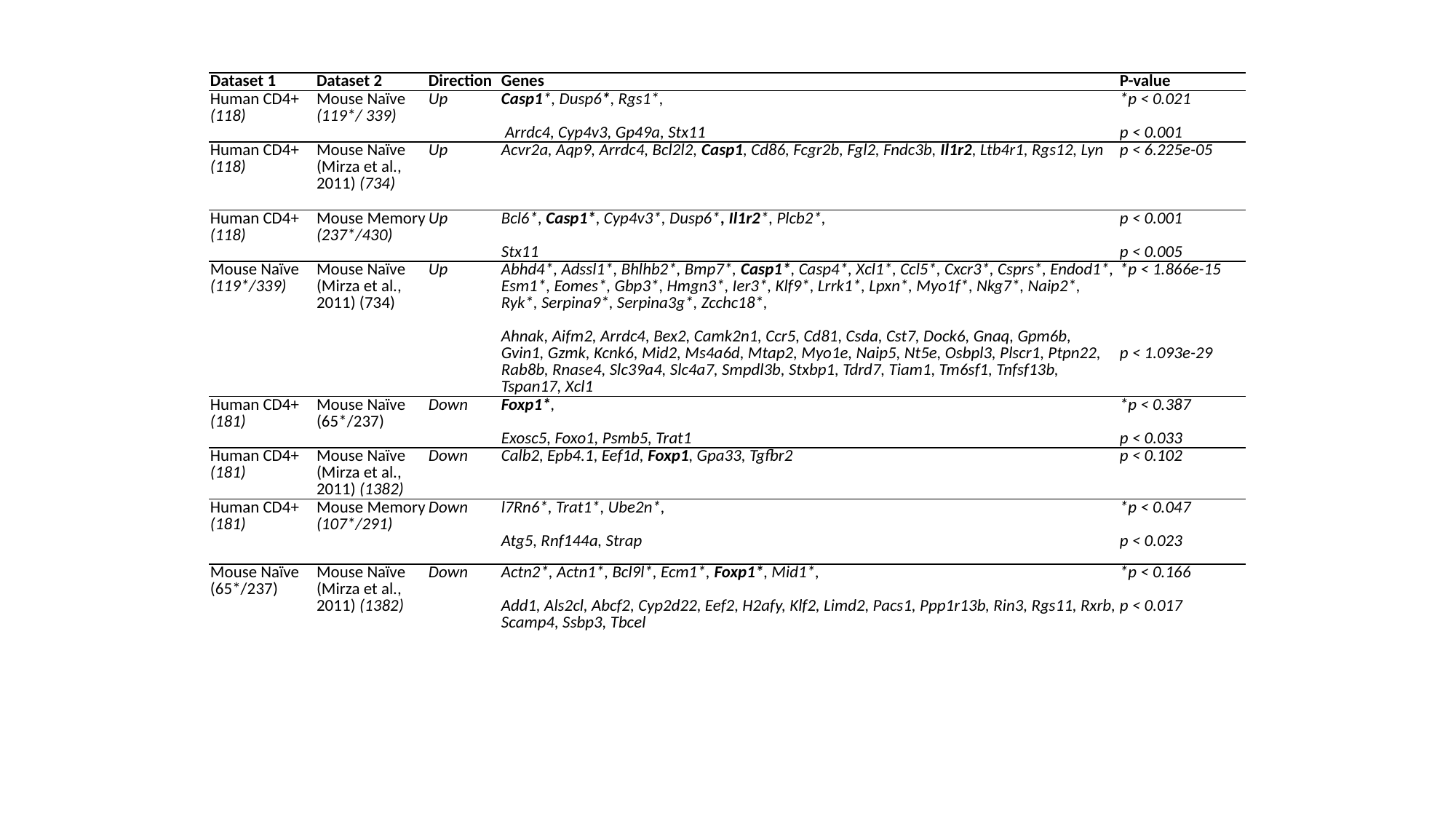

| Dataset 1 | Dataset 2 | Direction | Genes | P-value |
| --- | --- | --- | --- | --- |
| Human CD4+ (118) | Mouse Naïve (119\*/ 339) | Up | Casp1\*, Dusp6\*, Rgs1\*, Arrdc4, Cyp4v3, Gp49a, Stx11 | \*p < 0.021 p < 0.001 |
| Human CD4+ (118) | Mouse Naïve (Mirza et al., 2011) (734) | Up | Acvr2a, Aqp9, Arrdc4, Bcl2l2, Casp1, Cd86, Fcgr2b, Fgl2, Fndc3b, Il1r2, Ltb4r1, Rgs12, Lyn | p < 6.225e-05 |
| Human CD4+ (118) | Mouse Memory (237\*/430) | Up | Bcl6\*, Casp1\*, Cyp4v3\*, Dusp6\*, Il1r2\*, Plcb2\*, Stx11 | p < 0.001 p < 0.005 |
| Mouse Naïve (119\*/339) | Mouse Naïve (Mirza et al., 2011) (734) | Up | Abhd4\*, Adssl1\*, Bhlhb2\*, Bmp7\*, Casp1\*, Casp4\*, Xcl1\*, Ccl5\*, Cxcr3\*, Csprs\*, Endod1\*, Esm1\*, Eomes\*, Gbp3\*, Hmgn3\*, Ier3\*, Klf9\*, Lrrk1\*, Lpxn\*, Myo1f\*, Nkg7\*, Naip2\*, Ryk\*, Serpina9\*, Serpina3g\*, Zcchc18\*, Ahnak, Aifm2, Arrdc4, Bex2, Camk2n1, Ccr5, Cd81, Csda, Cst7, Dock6, Gnaq, Gpm6b, Gvin1, Gzmk, Kcnk6, Mid2, Ms4a6d, Mtap2, Myo1e, Naip5, Nt5e, Osbpl3, Plscr1, Ptpn22, Rab8b, Rnase4, Slc39a4, Slc4a7, Smpdl3b, Stxbp1, Tdrd7, Tiam1, Tm6sf1, Tnfsf13b, Tspan17, Xcl1 | \*p < 1.866e-15 p < 1.093e-29 |
| Human CD4+ (181) | Mouse Naïve (65\*/237) | Down | Foxp1\*, Exosc5, Foxo1, Psmb5, Trat1 | \*p < 0.387 p < 0.033 |
| Human CD4+ (181) | Mouse Naïve (Mirza et al., 2011) (1382) | Down | Calb2, Epb4.1, Eef1d, Foxp1, Gpa33, Tgfbr2 | p < 0.102 |
| Human CD4+ (181) | Mouse Memory (107\*/291) | Down | l7Rn6\*, Trat1\*, Ube2n\*, Atg5, Rnf144a, Strap | \*p < 0.047 p < 0.023 |
| Mouse Naïve (65\*/237) | Mouse Naïve (Mirza et al., 2011) (1382) | Down | Actn2\*, Actn1\*, Bcl9l\*, Ecm1\*, Foxp1\*, Mid1\*, Add1, Als2cl, Abcf2, Cyp2d22, Eef2, H2afy, Klf2, Limd2, Pacs1, Ppp1r13b, Rin3, Rgs11, Rxrb, Scamp4, Ssbp3, Tbcel | \*p < 0.166 p < 0.017 |
